# Dissipation dynamics of fipronil in tropical paddy (*Oryza sativa L.*) soil: unravelling its persistence and metabolism under varying moisture regime

**DOI:** 10.1186/s12870-026-09282-3

**Published:** 2026-06-23

**Authors:** D C Preethu, K R Ashoka, T Bhagyalakshmi, S B Yogananda, L Vijaykumar, N Kirankumar, Manjanagouda S Sannagoudar

**Affiliations:** 1 University of Agricultural Sciences, Bangalore, Karnataka 560065 India; 2 University of Agricultural Sciences, Mandya, Karnataka 571405 India; 3 ICAR-National Institute of Seed Science and Technology, Regional Station, GKVK Campus, Bangalore, Karnataka, 560065 India

**Keywords:** Moisture regime, Organic matter amendment, metabolites, dissipation, half life

## Abstract

**Background:**

Fipronil, a phenylpyrazole insecticide, is widely used for the management of rice pests; however, its environmental fate and potential risks in tropical paddy ecosystems to food safety under varying soil conditions remain insufficiently understood.

**Methods:**

A field study was conducted in a tropical paddy ecosystem with sandy loam soil to evaluate the uptake, translocation, and dissipation dynamics of fipronil under different soil moisture regimes (flooded, saturated, and field capacity) with and without organic matter amendment. Fipronil and its metabolites in soil and plant samples were quantified using LC–MS/MS following the QuEChERS extraction method validated using standard method validation parameters.

**Results:**

Fipronil applied at 75 g a.i. ha⁻¹ (0.3G formulation) at 20 days after transplanting showed a progressive decline in soil residues from 0.70, 0.79, and 1.37 µg g⁻¹ (day 1) to 0.01, 0.02, and 0.03 µg g⁻¹ (30 DAA) under flooded, saturated, and field capacity conditions, respectively, indicating faster dissipation under flooded conditions. The dissipation of fipronil was 1.10% and 0.76% higher under flooded than saturated and field capacity conditions respectively. Organic matter-amended Fipronil soils showed higher persistence, with half-lives of 11.87 days (flooded without organic matter) to 13.75 days (field capacity with organic matter) in cropped soils and 20.82 days in non-cropped soil, indicating prolonged residue retention and potential environmental risks. Plant uptake was rapid, peaking at 2 days after application, followed by a gradual decline. Residue levels in plants were lower under organic amendment, indicating reduced bioavailability. Fipronil predominantly accumulated in roots, followed by stem and leaves, facilitating translocation to aerial parts. Among metabolites, Fipronil sulfone dominated, reaching 0.82 µg g⁻¹ in field capacity, 0.63 µg g⁻¹ in saturated, and 0.60 µg g⁻¹ in flooded soils at 15 DAA, indicating oxidative persistence and higher toxicity. Sulfide (0.10 µg g⁻¹ at 15 DAA), reflecting anaerobic degradation, while desulfinyl remained below 0.01 µg g⁻¹. Harvest residues remained below MRL (< 0.01 µg g⁻¹), indicating low health risk.

**Conclusions:**

These findings provide important scientific evidence for developing irrigation- and organic amendment-based pesticide management strategies to support safer fipronil use, residue regulation, and sustainable rice production in tropical paddy ecosystems.

## Introduction

Rice (*Oryza sativa* L.) is a fundamental staple crop that sustains over half of the global population, with India leading as the world’s second-largest producer and maintaining the largest area under paddy cultivation [1]. However, paddy cultivation faces significant challenges from diverse pest complexes that can cause substantial yield losses [2], necessitating effective pest management strategies to ensure food security and economic sustainability.

Pesticide application has become indispensable for managing agricultural pests and diseases, yet the continuous use of various pesticides in cropping systems raises concerns about residue accumulation in agricultural soils. These residues can be absorbed by crop roots and subsequently translocated to edible plant parts, creating persistent risks to food safety and human health [3]. Understanding the environmental fate and behavior of pesticides under different agricultural conditions is therefore crucial for developing sustainable pest management practices that balance efficacy with safety.

Understanding the fate and behavior of fipronil under tropical conditions is essential for evaluating its dissipation patterns under different moisture regimes and environmental conditions. Paddy cultivation represents one of the most important agricultural systems in tropical regions of Asia and Africa, characterized by unique flooded soil conditions that create distinct physicochemical environments. The tropical paddy agro-ecosystem presents a complex interplay of factors including soil characteristics such as organic matter content, clay content, and moisture levels, all of which are key determinants of fipronil persistence and transformation [4].

The unique characteristics of paddy systems particularly the alternating aerobic and anaerobic conditions, variable moisture regimes, and potential for organic matter amendments create specific environmental contexts that may significantly influence fipronil fate and behaviour [4]. However, systematic studies examining these interactions under controlled field conditions in tropical environments are scarce, limiting our ability to develop evidence-based recommendations for sustainable fipronil use in rice production systems.

Fipronil is a systemic, broad-spectrum phenylpyrazole insecticide [5-amino-3-cyano-1-(2,6-dichloro-4-trifluoromethylphenyl)-4-trifluoromethyl sulfinyl pyrazole] confers unique properties that make it particularly effective against a wide range of insect pests such as rice stem borer, leaf folder. It acts as a contact and stomach poison by blocking GABA-gated chloride channels in insects, causing paralysis and death [2].

Furthermore, its distinct mode of action makes fipronil particularly valuable for managing insect pests that have developed resistance to conventional insecticides, including pyrethroids (sodium channel blockers), organophosphates, and carbamates (cholinesterase inhibitors) [2]. The availability of fipronil as a 0.3GR granular formulation facilitates convenient application through broadcasting over standing paddy crops, making it operationally suitable for rice cultivation systems.

The environmental fate of fipronil is characterized by multiple transformation pathways that are strongly influenced by prevailing environmental conditions. Under aerobic conditions, fipronil readily undergoes oxidation reactions, converting to fipronil sulfone as the primary metabolite, whereas high moisture content or anaerobic conditions promote reduction reactions leading to fipronil sulfide formation [4]. Additionally, alkaline soil conditions can trigger hydrolysis reactions that produce fipronil amide, while exposure to high-intensity sunlight initiates photolysis processes resulting in the formation of fipronil-desulfinyl [5].

The toxicological profile of fipronil metabolites presents significant environmental and ecological concerns, as these transformation products often exhibit greater toxicity than the parent compound. The sulfone metabolite demonstrates 6.3 times higher toxicity to rainbow trout and 3.3 times greater toxicity to bluegill sunfish compared to fipronil itself. Similarly, fipronil shows pronounced toxicity toward freshwater aquatic invertebrates, with the sulfone metabolite being 6.6 times more toxic and the desulfinyl photodegradate exhibiting 1.9 times higher acute toxicity than the parent compound [6]. These findings underscore the importance of understanding not only parent compound behavior but also the formation and persistence patterns of metabolites in agricultural environments.

The persistence of fipronil in soil environments is governed by multiple interacting factors, including soil moisture, pH, organic matter content, temperature, sunlight intensity, and microbial population dynamics [7, 8]. The half-life of fipronil exhibits considerable variability, ranging from 122 to 128 days under controlled laboratory conditions and extending from 3 to 7.3 months under field conditions [9]. This variability highlights the complex nature of pesticide-environment interactions and the need for site-specific understanding of degradation processes.

Organic amendments can significantly alter fipronil fate and behavior in soil systems, though the direction and magnitude of these effects depend on the specific amendment type and environmental conditions [8]. demonstrated this complexity in studies conducted on soils from Mato Grosso do Sul, Brazil, where the application of vinasse reduced fipronil persistence (half-life of 15 days) and enhanced degradation, whereas filter cake addition decreased degradation rates (half-life of 105 days) by increasing fipronil sorption to soil particles. These contrasting effects illustrate the importance of understanding how different organic amendments interact with pesticide molecules under varying environmental conditions.

Although several studies have reported the persistence and degradation behavior of Fipronil in agricultural soils, most investigations have been confined to controlled laboratory conditions or conventional flooded rice systems and primarily focused on the dissipation of the parent compound. Limited information is available on the influence of varying soil moisture regimes, organic matter amendments, crop-mediated effects, plant uptake, translocation patterns, and metabolite formation under tropical paddy ecosystems. Regional climatic conditions further exert profound influences on pesticide dissipation patterns, with tropical environments generally promoting faster degradation due to elevated temperatures, enhanced microbial activity, and accelerated biodegradation processes. However, despite the widespread use of Fipronil in tropical agriculture, comprehensive information on its behavior under representative tropical paddy soil conditions remains inadequate.

Therefore, the present study was undertaken to address these research gaps by comprehensively evaluating the dissipation dynamics of Fipronil in press mud compost-amended and herbicide-applied paddy soils under three distinct moisture regimes, namely flooded, saturated, and field capacity conditions in a tropical field environment with the following objectives: (i) to determine the persistence and dissipation kinetics of Fipronil under varying moisture regimes and assess the influence of organic matter amendment on residue retention and degradation behavior; (ii) to identify and quantify major metabolites formed during degradation and evaluate plant uptake and translocation patterns in different rice plant parts; and (iii) to assess residue levels at harvest and their implications for food safety in tropical rice production systems.

## Materials and methods

### Analytical chemicals and reagents:

 The certified reference materials (CRM) of fipronil (purity of 99.2% Batch no: RDL134-1-10; Batch code: AE F124964-PU-01; cert no: AZ 21491) and its three major metabolites desulfinyl (purity 98.5%; Batch no: 10DGM22; Batch code: AE 0592168 00 1B98 001; cert no: AZ 21433), sulfide (purity 98.0%; Batch no: 893008; Batch code: AE F124966 00 1B99 0001; cert no: AZ 17983) and sulfone (purity 99.7%; Batch no: CBL 15; Batch code: AE F126098-PU-01; cert no: AZ 16096) were procured from Bayer Crop Science limited, Maharashtra, India. The commercial formulation of fipronil 0.3% GR (trade name: REGENT- Fipronil 0.3GR at 75 g a.i. ha⁻¹) was procured from Adama India Pvt. Ltd., Hyderabad for field application in paddy cultivation. High purity solvents i.e. ethyl acetate and acetonitrile (purity 99.9%, J.T. Baker, USA) were used for extraction and residue analysis. Ultra-pure water was generated using an Evoqua ultra-pure water purification system. Analytical reagent such as magnesium sulphate heptahydrate (Loba Chemie Pvt. Ltd.), anhydrous sodium sulphate (Merck, India), sodium acetate (Isochem Laboratories, India), acetic acid (Merck, India), LC-MS grade methanol (Merck, India) were employed during sample preparation. For clean-up procedure, sorbent including Primary Secondary Amine (PSA; 40 μm, Bondesil, Agilent, USA), octadecylsilane (C18, Agilent, USA) and graphitized carbon black (GCB; Loba Chemie Pvt. Ltd.) were used in residue analysis.

Stock standard solutions: CRMs of fipronil, fipronil-sulfone, fipronil-sulfide and desulfinyl were taken from the refrigerator (-15 °C) and kept for 1 h until the CRM attained to room temperature. The amount of CRM transferred to prepare primary stock solution was given in below.

**Table:** Weight of CRM, per cent purity and standard stock concentration

Sl No	Name of CRM	Weight of CRM (mg)	Per cent purity	Stock concentration (µg mL^− 1^)
1	Fipronil	10.2	99.2	1011.84
2	Fipronil Sulfone	12.0	99.7	1196.40
3	Fipronil Sulfide	11.2	98.0	1097.60
4	Fipronil Desulfinyle	12.2	98.5	1201.70

Further, CRM was dissolved with approximately 2–3 mL of LC-MS grade methanol and made the volume up to 10 mL. The final concentration was calculated using formulae given below.

$$Concentration\;\left(\mu gml^{-1}\right)=\frac{Weight\;of\;CRM\left(mg\right)}{Final\;Volume\;\left(ml\right)}\times\frac{Purity\;of\;CRM\;\left(\%\right)}{100}\times100$$

### Intermediate standard solution

An intermediate solution having concentration of 1000 µg ml^− 1^ was prepared separately, from primary stock solution volume of 9.88, 8.36, 9.11 and 8.32 mL for fipronil, fipronil sulfone, fipronil sulphide and fipronil desulfinyle respectively and was made (10 ml) with LC-MS grade methanol and the solution was stored at -15 °C.

Working standard solutions for fortification and quantification were subsequently prepared by appropriate dilution of the stock solution with methanol. All prepared standards and samples were stored at − 20 °C until further use. The stability of these compounds in methanol at − 20 °C was evaluated in the present study and was found to be stable for up to twelve months. According to [10], these compounds exhibit stability in methanol of a period more than 15 months at -20 °C.

#### Field study

**Fig. 1:**
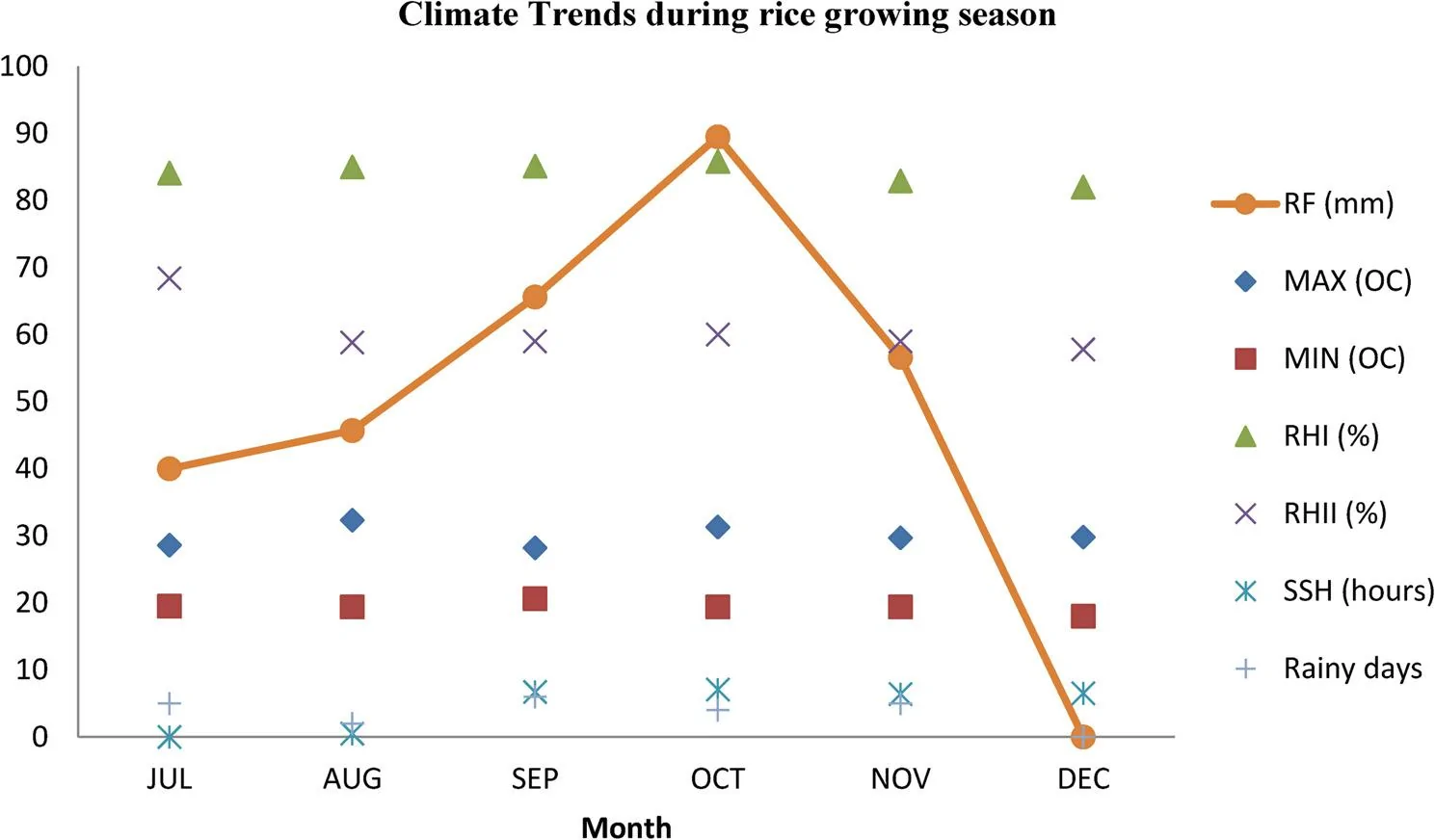
Climate trends during rice growing season at College of Agriculture, V C Farm Mandya

A supervised field experiment of fipronil 0.3% GR was conducted on short duration (125 days) variety of paddy viz., RNR 15048 following split plot design at VC Farm, Mandya District, Karnataka State, India. Experiment was conducted in a paddy field where paddy was grown for the past 10 years without the application of fipronil. During paddy growing period (July to December 2023 &2024) total rain fall received was 542.4 mm (October month with highest RF of 89.5 mm) with 33 rainy days, average temperature ranged from 22 to 33 °C, RH was 84.15% and 4.53 sunshine hours (Fig. 1). The soil at experiment site was sandy clay loam in texture with sand 50.3%, silt − 19.5% and clay − 30.2%) belongs to *Alfisol* (*Typic rhodustalfs*) according to United States Department of Agriculture. Soil had low bulk density 1.32 gcc^− 1^, 23.66% Water Holding Capacity (WHC). Soil was alkaline in pH (8.38), medium in organic carbon content (0.60%), normal soluble salt content (0.28 dS m^− 1^) and medium cation exchange capacity (17.7 cmol (p^+^) kg^− 1^ soil) with medium in available nitrogen (401.40 kg ha^− 1^), P_2_O_5_ (53.60 kg ha^− 1^) and K_2_O (175.66 kg ha^− 1^), exchangeable calcium (5.8 me/100 g), exchangeable magnesium (2.6 me/100 g) and exchangeable sodium (3.8 me/100 g) level. The soil samples collected from the experimental site were analyzed prior to the study and were found to be free from fipronil residues.

The pressmud applied as a soil amendment was characterized for its physical and chemical properties using standard analytical procedures. The analysis revealed that pressmud was nearly neutral in reaction (pH 7.74) and contained 35.65% total organic carbon. It also had 1.28% total nitrogen, resulting in a C: N ratio of 27.85. The total nutrient content comprised 3.3% phosphorus, 1.82% potassium, 2.5% calcium, 1.0% magnesium, and 1.20% sulphur. In addition, the micronutrient concentrations were 115 ppm zinc, 80 ppm copper, 400 ppm iron, and 450 ppm manganese.

Experiment was conducted with nine treatments (Table 1) replicated thrice. Each experimental sub plots measured 3mX3m (9m^2^) for flooded (FL), saturated (ST), field capacity (FC) moisture regime with organic unamend, amended and herbicide application. A distance of 0.5 m was set to differentiate vertical factors consisting of without organic amendment, Organic amendment and herbicide interaction and 1 m was set to differentiate horizontal factors comprised of three different moisture regimes by raising the bund height of about 30 cm in the space available between plots.

Press mud compost was used as organic amendment for the T_4_ to T_6_ treatments. Pressmud compost was thoroughly mixed with the soil at 10t ha^-1^ which corresponds to the recommended dose of farmyard manure for paddy fields. Combination of two active ingredients, bensulfuron-methyl (0.6%) and pretilachlor (6%) (herbicide is a pre-emergence herbicide widely used in rice cultivation to control a broad spectrum of weeds) was broadcasted to study the effect of pesticide interaction on fipronil degradation for T_7_ to T_9_ treatments at 10 kg ha^-1^ which corresponds to the recommended dose of herbicide for paddy fields. T_1_ to T_3_ treatments are without organic amendment and herbicide. Herbicide interaction was studied based on the hypothesis that simultaneous application of multiple agrochemicals in paddy fields may influence fipronil degradation in soil. Herbicide residues can alter soil microbial activity and chemical transformation processes, affecting fipronil persistence under realistic field application conditions.

Commercial grade Fipronil 0.3GR at 75 g a.i. ha⁻¹ was added to each plot which corresponds to the recommended dose of fipronil for paddy fields was applied at the tillering stage (20 days after transplanting – DAT). The moisture content of soil was maintained to maintain different moisture regime. For flooded conditions, a standing water depth of 3–5 cm was maintained continuously above the soil surface throughout the experiment. Under saturated conditions, soil moisture was maintained at approximately 90–100% of water holding capacity (WHC), ensuring continuous wetness without standing water. For field capacity conditions, soil moisture was maintained at approximately 60–70% of WHC through periodic irrigation whenever thin cracks began to appear on the soil surface, ensuring the soil stayed moist.

After fipronil application, the paddy plant, cropped soil and no cropped soil (control-CT) representative samples (0–15 cm) were drawn using soil auger from each plot at 0 (2 h after application), 1, 3, 5, 10, 15, 30, 50, 60 and 70 days after the application (DAA) of fipronil pesticide by following the standard sampling procedure. Control (CT) plot was the undisturbed plot without the paddy crop which received recommended dose of fipronil. Paddy whole grain and plant parts (roots, straw and leaves) were collected and analysed for fipronil and its metabolites content on fresh weight basis. Collected samples were transported to the laboratory in an ice packed box for pesticide residue analysis. The samples were freeze-stored prior to extraction. The residue of fipronil in soil samples and plant samples were analysed using LCMSMS by following QuChERS method.

**Table 1:** Experimental treatments

T_1_:	Flooded condition without organic matter (FL + WOM)
T_2_:	Saturated condition without organic matter (ST + WOM)
T_3_:	Field capacity condition Without organic matter (FC + WOM)
T_4_:	Flooded condition with organic matter -Pressmud compost (FL + OM)
T_5_:	Saturated condition with organic matter -Pressmud compost (ST + OM)
T_6_:	Field capacity condition with organic matter -Pressmud compost (FC + OM)
T_7_:	Flooded condition with herbicide-Bensulfuron methyl + Pretilachlor (FL + WD)
T_8_:	Saturated condition with herbicide-Bensulfuron methyl + Pretilachlor (ST + WD)
T_9_:	Field capacity condition with herbicide-Bensulfuron methyl + Pretilachlor (FC + WD)
T_10_:	Control- undisturbed plot without crop (CT)

#### Soil redox potential under different moisture regime

Redox potential (Eh) was monitored in each treatment at 0 (2 h after the second application), 1, 3, 5, 10, 15, 30, 50, 60, and 70 days after application (DAA) in the paddy field. Measurement was carried out using combined waterproof ORP/redox meter (Eutech Instruments, USA) by inserting the probe directly into soil and recording the potential difference in millivolts (mV) following standard procedures [11]. Observations were recorded at multiple locations around the flux measurement setup to represent conditions from the rhizosphere to the bulk soil interface.

### QuEChERS-based extraction and LC–MS/MS analysis of fipronil residues in soil and paddy plant

Fipronil residues in paddy soil and plant were extracted using a modified QuEChERS (Quick, Easy, Cheap, Effective, Rugged and Safe) method to improve analyte recovery and minimize matrix interference associated with these complex matrices such as high moisture soil and paddy plants, as the original protocol was designed for fruits and vegetables. Acetonitrile was selected as the extraction solvent for efficient recovery of moderately non-polar fipronil while minimizing the co-extraction of lipids and pigments compared to solvents such as acetone or ethyl acetate. Ten grams soil/ grounded plant samples were taken in triplicates in teflon centrifuge tubes. Acetonitrile (10 ml), 6 g anhydrous sodium sulphate and 1.5 g sodium acetate were added and vortexed followed by shaking on a rotospin for 15 min at 50 rpm. The samples were centrifuged at 5000 rpm for 5 min and 2 ml of the supernatant was transferred for clean-up into 5 ml eppendorf tubes containing dispersive Solid Phase Extraction (d-SPE) sorbents viz., 150 mg magnesium sulphate, 100 mg Primary Secondary Amine (PSA). Graphitized carbon black (GCB) 15 mg was added for pigmented samples. After vortexing and centrifugation (for 5 min at 10000 rpm), the extract was filtered through a 0.2 μm nylon filter and transferred into the vials for analysis in LCMSMS. Modifications were validated through recovery studies. Recovery studies were performed by spiking blank soil and paddy plant with fipronil standards (5–100 µg kg⁻¹). The matrix effect was calculated using the following equation: ME%= (Peak area of matrix standard- Peak area of solvent standard)/ Peak area of solvent standard *100 (European Commission, 2019). Procedural blanks and quality control samples were included in each batch of analysis to ensure the accuracy, precision, and reliability of the analytical method. Matrix-matched calibration standards were used for quantification to minimize ion suppression/enhancement during LC-MS/MS analysis.

#### LC–MS/MS instrumental conditions for analysis

All samples were analysed using a Shimadzu LCMS-8045 equipped with a triple quadrupole mass spectrometer (MS/MS). The chromatographic separation was performed using Shim pack GISS C18 column (100 mm×2.1 mm×3 μm particle size), consisting of octadecyl stationary phase supported on high purity porous spherical silica. The mobile phase comprised a 50:50 (v/v) mixture of methanol and water, delivered under isocratic conditions at a flow rate of 0.4 mL min⁻¹. Nitrogen was used as the collision and nebulizing gas, maintained at a constant flow rate of 3.0 L min⁻¹, ion source temperature of 375 °C, nebulizer pressure at 35 psi. and sample injection volume of 1 µL was employed for analysis. Detection was performed using negative electrospray ionization (ESI) mode, and quantification was performed using Multiple Reaction Monitoring (MRM). The optimized instrumental parameters for data acquisition are summarized in Table 2.

**Table 2:** Optimized LC–MS/MS Parameters for fipronil and its Metabolites

Analyte	Retention Time(mins)	Qualitative and Quantitative Ion transitation (m/z)	Fragmentor (V)	Collision energy (V)
Fipronil	8.27	435/330	120	8
Fipronil sulfide	8.32	419/382	130	8
Fipronil sulfone	8.23	451/415	130	8
Fipronil desulfinyl	8.42	387/350	100	8

### Calibration and method validation

Calibration linearity (R²) and analytical sensitivity were evluated by preparing mixed standard solutions of fipronil and its metabolites in solvent and matrix blank extracts and plotting calibration curve over a concentration range of 0.005–0.100 µg g^− 1^. The limit of detection (LOD) and limit of quantification (LOQ) was estimated as three and ten times respectively, the ratio of the average standard deviation of the peak area across all calibration levels to the slope of the calibration curve [10]. Method accuracy (trueness) and precision were determined through recovery studies by fortifying the blank matrix with the target analytes at concentration levels of 0.005, 0.010, and 0.100 µg g⁻¹, followed by analysis using the established procedure. Precision was evaluated in terms of intra-day repeatability and expressed as relative standard deviation (RSD).

#### Statistical analysis and kinetic modelling

Individual data sets on fipronil residues and associated soil parameters were subjected to statistical analysis using the IBM SPSS Statistics 27.0.1 software package. The dissipation behavior of fipronil was further evaluated by fitting nonlinear regression models using Microsoft Excel. The degradation followed first-order kinetics, described by the equation C=C_0_e^− kt^, where C is the chemical concentration (µg g^− 1^) at time t (days), C_0_ is the initial concentration (µg g^− 1^), and k is the first-order rate constant (d^− 1^), independent of C and C_0_. The goodness of fit between the observed data and the first-order kinetic model was evaluated using the coefficient of determination (R²).The dissipation of the compounds was expressed as DT_50_ (t_1/2_), the time required for 50% of the pesticide to degrade was calculated using Hoskins’ formula, (DT_50_= (ln2)/k).

## Results

### Method validation

Calibration curves for fipronil and its metabolites showed good linearity over the concentration range of 0.005–0.100 µg g⁻¹. The retention times of the analytes (Fipronil-8.42; Fipronil sulfone-8.32; Fipronil sulphide-8.23; Fipronil desulfinyl-8.27) were consistent throughout the analysis. Mean recoveries of fipronil and its metabolites from fortified paddy plant and soil samples ranged from 78.01% to 95.47%, with relative standard deviation (RSD) values between 3.55% and 12.92% (Table 3) which are well within the generally accepted analytical validation limit of < 20% for pesticide residue analysis. Both reagent-only and matrix-matched calibration curves exhibited good linearity (R² > 0.99) within the tested concentration range. Matrix effect values were within ± 10%, indicating minimal signal enhancement or suppression (Table 3). The limit of detection (LOD) for fipronil and its metabolites was 0.001 µg g⁻¹ each, while the limit of quantification (LOQ) was 0.005 µg g⁻¹ for all matrices.

### Soil dissipation

The dissipation of fipronil in tropical paddy soils was studied under different moisture regimes, organic amendment, and herbicide application over 70 days (Table 4). Residues declined progressively and reached below detectable limits by 50 DAA, except in non-cropped control soil, where residues persisted above the maximum residue limit until 60 DAA. All treatments recorded residues below the MRL after 15 DAA, although non-cropped soil showed the highest level (0.50 µg g⁻¹).

Among cropped soils, flooded conditions resulted in the lowest residues (0.06–0.08 µg g⁻¹), followed by saturated (0.09–0.11 µg g⁻¹) and field capacity soils (0.10–0.12 µg g⁻¹) at 15 DAA. Organic amendment enhanced dissipation, particularly under flooded conditions (92.16%), followed by saturated (91.43%) and field capacity soils (89.87%).

Saturated soils showed moderate dissipation, with slightly higher persistence under organic amendment. Field capacity conditions exhibited the slowest dissipation and highest persistence, especially with organic inputs. Herbicide treatments showed relatively faster dissipation than organic amendments, with moderate residue levels across all moisture regimes.

**Table 3:** Recovery results of fipronil and its metabolites in paddy plant and soil

		Fipronil			Sulfone			Sulfide			Desulfinyl		
Matrix	Spiking levelµg kg^− 1^	R%	%RSD	%ME	R%	%RSD	%ME	R%	%RSD	%ME	R%	%RSD	%ME
Soil	5	86.40	7.23	-18.66	87.64	7.46	-6.76	91.80	4.73	-4.34	89.60	10.66	-4.87
Root		81.71	6.68	-15.56	81.59	6.08	-14.56	82.55	8.43	-7.34	82.22	7.61	-4.98
Straw		88.22	5.73	-2.87	78.47	9.24	-6.34	78.47	9.24	-4.87	82.30	7.56	-5.37
Leaf		83.98	5.61	-9.54	80.22	9.51	-6.77	80.22	9.51	-11.68	81.79	7.36	-9.44
Grain		85.51	7.51	-6.45	83.14	10.04	-3.67	83.14	10.04	-7.23	84.51	8.63	-15.21
Soil	10	90.50	5.99	-16.45	87.59	4.12	-11.54	87.56	8.03	-16.76	87.73	10.10	-11.45
Root		83.06	4.98	-5.45	84.02	9.06	-7.54	78.01	3.65	-7.45	85.39	3.63	-8.48
Straw		88.17	3.55	-11.45	86.44	12.92	-12.67	86.44	12.92	-9.51	84.53	4.36	-4.22
Leaf		89.54	5.70	-13.56	85.37	5.38	-12.87	85.37	5.38	-5.92	85.59	8.78	-7.34
Grain		81.67	4.20	-12.65	83.20	5.55	-15.65	83.20	5.55	-15.92	87.91	7.23	-3.65
Soil	100	95.47	4.99	-4.56	89.49	7.46	-8.56	92.72	3.87	-2.54	84.58	11.65	-5.91
Root		85.43	6.55	-8.56	87.44	9.86	-3.54	93.38	6.13	-9.49	84.40	6.55	-2.88
Straw		88.12	5.24	-5.65	87.99	6.23	-4.55	87.99	6.23	-9.77	84.25	6.60	-8.32
Leaf		86.13	5.48	-4.65	85.49	6.75	-8.45	85.49	6.75	-7.89	86.50	8.20	-1.78
Grain		85.41	5.97	-3.78	85.08	10.56	-1.88	85.08	10.56	-3.85	87.33	5.31	-7.33

Notation: Data of three replicates (*n* = 3); R=Mean Recovery RSD = Relative Standard Deviation (intra-day repeatability check); ME = Matrix Effect

**Table 4:** Persistence and dissipation kinetics of fipronil in paddy soil under different moisture regime, organic amendment and herbicide

DAA	Residue level (µg g^− 1^)										
	WOM			OM			WD			Control	*R* ^2^
	FL	ST	FC	ST	FC	FL	ST	FC			
0	0.70 ^a^	1.04 ^b^	1.11 ^b^	1.02 ^b^	1.62 ^d^	1.68 ^de^	0.79 ^a^	1.37 ^c^	1.48 ^cd^	1.86 ^e^	0.92
1	0.65 ^a^	0.96 ^b^	1.04 ^b^	0.95 ^b^	1.51 ^de^	1.59 ^ef^	0.74 ^a^	1.28 ^c^	1.42 ^cd^	1.69 ^f^	0.94
3	0.61 ^a^	0.94 ^b^	0.99 ^b^	0.92 ^b^	1.41 ^de^	1.50 ^e^	0.72 ^a^	1.19 ^c^	1.35 ^d^	1.67 ^f^	0.95
5	0.48 ^a^	0.69 ^b^	0.78 ^bc^	0.72 ^b^	1.05 ^d^	1.07 ^d^	0.57 ^a^	0.83 ^c^	1.05 ^d^	1.20 ^e^	0.94
10	0.23 ^a^	0.34 ^bc^	0.39 ^cd^	0.34 ^bc^	0.43 ^de^	0.46 ^e^	0.30 ^b^	0.40 ^cde^	0.43 ^de^	0.83 ^f^	0.95
15	0.06 ^a^	0.09 ^abc^	0.10 ^bcd^	0.08 ^ab^	0.11 ^de^	0.12 ^e^	0.08 ^ab^	0.10 ^cde^	0.11 ^de^	0.50 ^f^	0.99
30	0.011^a^	0.016^abc^	0.024 ^bcd^	0.024 ^ab^	0.025 ^e^	0.027 ^e^	0.021 ^ab^	0.028 ^cde^	0.030 ^de^	0.085 ^f^	0.98
50	0.00 ^a^	0.00 ^a^	0.00 ^a^	0.00 ^a^	0.00 ^a^	0.00 ^a^	0.00 ^a^	0.00 ^a^	0.00 ^a^	0.06 ^b^	1.00
60	BDL	BDL	BDL	BDL	BDL	BDL	BDL	BDL	BDL	BDL	-
70	BDL	BDL	BDL	BDL	BDL	BDL	BDL	BDL	BDL	BDL	-
R^2^	0.98	0.98	0.98	0.98	0.98	0.98	0.98	0.98	0.99	0.94	
K	0.058	0.056	0.055	0.056	0.052	0.05	0.058	0.053	0.052	0.033	
t_1/2_	11.87	12.42	12.6	12.29	13.38	13.75	11.95	13.05	13.3	20.82	

*FL* Flooded, *ST* Saturated, *FC* Field Capacity, *WOM *Without organic amendment, *OM *Organic amendment, *WD*-herbicide application, *BDL* Below Detectable Limit; non cropped undisturbed soilMean of three replicate observations. In a column, means followed by a common letter are not significantly different (*P* < 0.05) by Duncan’s multiple range test

## Fipronil metabolites

Among the metabolites, formation of sulfone metabolite was predominant metabolite, contributing nearly 40–60% higher residue levels than sulfide under field capacity and saturated conditions at later stages of dissipation. Formation of sulfone metabolite (Table 5) was significantly more in FC and saturated soils (FC-0.14, 0.09, 0.12 and ST-0.07, 0.05, 0.06 µg g⁻¹ at 60 DAA under WOM, OM and WD respectively) compared to flooded soil (0.04, 0.05, 0.04 µg g⁻¹ at 60 DAA under WOM, OM and WD respectively). Sulfone metabolite formation peaks even at 70 DAA with the highest residue observed in FC soil. Lower accumulation under Flooded and organic amended soil showed reduced oxidation of fipronil in anaerobic conditions. Sulfide metabolite (Table 5) accumulate significantly more in flooded soil (0.10 µg g⁻¹ at 15 DAA), suggesting anaerobic degradation pathways dominate in flooded environments. FC and saturated soil have significantly lower sulfide metabolite levels, indicating limited anaerobic activity. Residue levels gradually reduced over the time.

Desulfinyl metabolite formation was detected below the maximum residue limit (0.01 µg g⁻¹) in all the treatments (Table 5). The residue was most pronounced under field capacity moisture regimes and in control conditions. Flooded anaerobic environments, particularly with organic amendment or herbicide, suppressed desulfinyl metabolite accumulation. Statistical analysis confirms significant differences between treatments.

**Fig. 2:**
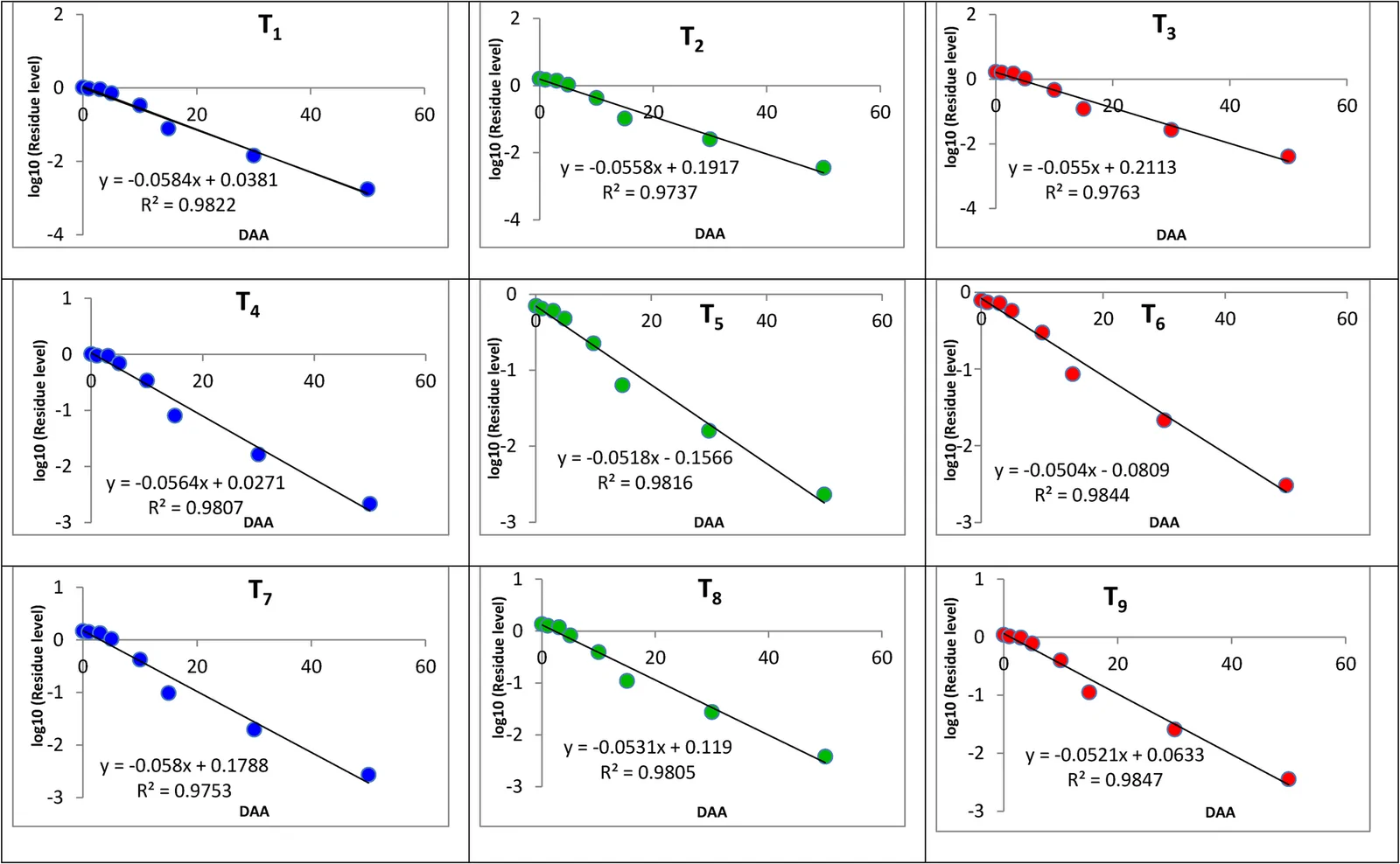
First order dissipation of fipronil in paddy soil under different moisture regimes with organic amendment and herbicide

Fipronil degradation data obtained from tropical paddy soil under different moisture regime, organic amendment and herbicide was fitted to the first order kinetics equation (Fig. 2). The amount of fipronil remaining in the soil under field experiment was plotted on a logarithmic scale against the incubation time. The degradation of fipronil in paddy soil followed a first-order reaction, as indicated by the linear plots based on the equation: C = C_0_e^-kt^. The half-life (t_1/2_) values (Table 4) were calculated using the formula: t_1/2_ = ln2/k. The results showed that the shortest half-life of 11.87 days was observed in flooded soil with organic unamended soil indicated the faster dissipation of fipronil with highest dissipation rate constant of 0.058(k). Longest half-life (13.75 days) in FC moisture soil with organic amendment indicated the longer persistence of fipronil with lowest dissipation rate constant (k) of 0.05. No cropped soil recorded longest half-life of 20.82 days of with dissipation rate constant of 0.033. A regression coefficient (R²) value more than 0.94 confirmed a strong linear relationship between time and fipronil dissipation.

**Table 5:** Residue level of fipronil metabolites in paddy soil under different moisture regime, organic amendment and herbicide

	DAA	Residue level (µg g^− 1^)										
		WOM			OM			WD			Control	*R* ^2^
		FL	ST	FC	FL	ST	FC	FL	ST	FC		
Sulfone	0	0.00 ^a^	0.00 ^a^	0.03 ^d^	0.00^a^	0.02 ^c^	0.04 ^f^	0.00 ^a^	0.01 ^b^	0.03 ^d^	0.03 ^e^	0.99
	1	0.01 ^a^	0.02 ^b^	0.04 ^c^	0.01 ^a^	0.03 ^c^	0.06 ^e^	0.01 ^a^	0.02 ^b^	0.05 ^d^	0.08 ^f^	0.97
	5	0.04 ^a^	0.13 ^b^	0.14 ^b^	0.05 ^a^	0.14 ^b^	0.16 ^c^	0.04 ^a^	0.13 ^b^	0.16 ^c^	0.19 ^d^	0.98
	15	0.50 ^a^	0.60 ^ab^	0.66 ^b^	0.60 ^ab^	0.63 ^b^	0.82 ^c^	0.52 ^a^	0.60 ^ab^	0.79 ^c^	0.98 ^d^	0.88
	50	0.18 ^a^	0.20 ^ab^	0.24 ^c^	0.20 ^ab^	0.24 ^c^	0.35 ^d^	0.19 ^ab^	0.22 ^bc^	0.33 ^d^	0.41 ^e^	0.94
	70	BDL	BDL	BDL	BDL	BDL	BDL	BDL	BDL	BDL	BDL	-
Sulfide	0	0.03 ^d^	0.01 ^b^	0.01 ^a^	0.03 ^de^	0.02 ^c^	0.02 ^a^	0.03 ^d^	0.02 ^c^	0.01 ^a^	0.03 ^e^	0.97
	1	0.03 ^d^	0.02 ^b^	0.01 ^a^	0.03 ^d^	0.02 ^c^	0.01 ^a^	0.03 ^d^	0.02 ^bc^	0.01 ^a^	0.03 ^d^	0.95
	5	0.05^d^	0.04 ^c^	0.02 ^a^	0.04^d^	0.04 ^cd^	0.03 ^b^	0.04^d^	0.04 ^cd^	0.02 ^a^	0.05^e^	0.92
	15	0.10 ^c^	0.04 ^b^	0.02 ^a^	0.10 ^c^	0.05 ^b^	0.03 ^a^	0.10^c^	0.04 ^b^	0.02 ^a^	0.10 ^c^	0.97
	50	0.02 ^e^	0.01 ^bcd^	0.01 ^a^	0.02 ^ef^	0.01 ^cd^	0.01 ^abc^	0.02 ^ef^	0.01 ^d^	0.01 ^ab^	0.02 ^f^	0.97
	70	BDL	BDL	BDL	BDL	BDL	BDL	BDL	BDL	BDL	BDL	-
Desulfinyl	0	0.00 ^a^	0.01 ^c^	0.01 ^c^	0.00 ^a^	0.01 ^b^	0.01 ^c^	0.00 ^a^	0.01 ^b^	0.01 ^c^	0.01 ^d^	0.95
	1	0.00 ^a^	0.01 ^c^	0.01 ^c^	0.00 ^a^	0.01 ^b^	0.01 ^c^	0.00 ^a^	0.01 ^b^	0.01 ^c^	0.01 ^d^	0.94
	5	0.01 ^b^	0.01 ^cd^	0.01 ^d^	0.01 ^a^	0.01 ^c^	0.01 ^cd^	0.01 ^a^	0.01 ^cd^	0.01 ^d^	0.02 ^e^	0.97
	15	0.01 ^a^	0.01 ^b^	0.03 ^c^	0.01 ^a^	0.01 ^b^	0.02 ^c^	0.01 ^a^	0.01 ^b^	0.02 ^c^	0.03 ^d^	0.97
	50	0.00 ^ab^	0.00 ^b^	0.01 ^e^	0.00 ^a^	0.00 ^ab^	0.01 ^c^	0.00 ^a^	0.00 ^b^	0.01 ^d^	0.01 ^f^	0.97
	70	BDL	BDL	BDL	BDL	BDL	BDL	BDL	BDL	BDL	BDL	-

*FL* Flooded, *ST* Saturated, *FC* Field Capacity, *WOM *Without organic amendment, *OM *Organic amendment, WD-herbicide application, *BDL *Below Detectable Limit; control-non cropped undisturbed soil; Mean of three replicate observations. In a column, means followed by a common letter are not significantly different (*P* < 0.05) by Duncan’s multiple range test

### Plant uptake

Fipronil exhibited uptake and translocation in paddy plants, with maximum residue concentrations observed at 2 DAA in roots, stems, and leaves (Tables 6, 7 and 8). Residue accumulation followed the order root > stem > leaf. In roots, residues increased from initial soil concentrations of 0.23–0.36 µg g⁻¹ soil (2 h after application) to 0.72–1.78 µg g⁻¹ plant at 2 DAA and gradually declined to 0.04–0.16 µg g⁻¹ by 30 DAA, reaching below detectable levels after 50 DAA. Flooded conditions recorded the highest root residues (0.08–0.16 µg g⁻¹ at 30 DAA), followed by saturated (0.07–0.13 µg g⁻¹) and field capacity conditions (0.04–0.08 µg g⁻¹). Organic-amended treatments recorded lower residues (0.04–0.09 µg g⁻¹) compared to unamended treatments (0.08–0.16 µg g⁻¹). 

Stem residues were lower than root residues, with peak concentrations of 0.40–0.44 µg g⁻¹ at 2 DAA and complete dissipation by 15 DAA under field capacity with organic amendment. Leaf residues were the lowest among plant parts, reaching maximum concentrations of 0.30–0.33 µg g⁻¹ at 2 DAA and dissipating completely after 5 DAA under field capacity with organic amendment. Among metabolites, sulfone residues remained low (0.02–0.03 µg g⁻¹) up to 3 DAA, peaked at 0.06–0.10 µg g⁻¹ between 10 and 30 DAA, and declined below MRL after 60 DAA. Sulfide residues reached maximum levels at 15 DAA and declined to below MRL levels (0.01–0.02 µg g⁻¹) after 60 DAA, while desulfinyl residues remained consistently low (0.01–0.02 µg g⁻¹) throughout the experimental period.

**Table 6:** Residue level of fipronil and its metabolites in paddy root under different moisture regime, organic amendment and herbicide

	DAA	Residue level (µg g^− 1^)									
		WOM			OM			WD			*R* ^2^
		FL	ST	FC	FL	ST	FC	FL	ST	FC	
Fipronil	1	0.36 ^e^	0.29 ^d^	0.28 ^d^	0.30 ^abc^	0.23 ^ab^	0.22 ^a^	0.31 ^d^	0.26 ^c^	0.25 ^bc^	0.89
	2	1.78 ^g^	1.19 ^d^	1.12 ^c^	1.24 ^e^	0.75 ^a^	0.72 ^a^	1.72 ^f^	1.18 ^d^	0.81 ^b^	0.99
	15	0.21 ^e^	0.12 ^c^	0.11 ^c^	0.17 ^d^	0.07 ^ab^	0.06 ^a^	0.18 ^d^	0.10 ^c^	0.08 ^b^	0.96
	50	0.09 ^d^	0.06 ^c^	0.06 ^c^	0.06 ^c^	0.03 ^b^	0.02 ^a^	0.08 ^d^	0.04 ^b^	0.03 ^b^	0.93
	60	BDL	BDL	BDL	BDL	BDL	BDL	BDL	BDL	BDL	-
Sulfone	1	0.01 ^g^	0.01 ^f^	0.01 ^ef^	0.01 ^b^	0.01 ^b^	0.01 ^a^	0.01 ^de^	0.01 ^cd^	0.01 ^c^	0.96
	2	0.01 ^f^	0.01 ^e^	0.01 ^d^	0.01 ^c^	0.01 ^b^	0.01 ^a^	0.01 ^d^	0.01 ^c^	0.01 ^c^	0.98
	15	0.10^e^	0.09^d^	0.08^c^	0.07 ^b^	0.07 ^ab^	0.07 ^a^	0.08^c^	0.08 ^bc^	0.07 ^b^	0.9
	50	0.05 ^e^	0.05 ^d^	0.05 ^d^	0.04 ^b^	0.04 ^a^	0.03 ^a^	0.04 ^c^	0.04 ^bc^	0.04 ^bc^	0.93
	60	0.05 ^g^	0.04 ^f^	0.04 ^e^	0.03 ^c^	0.03 ^b^	0.02 ^a^	0.04 ^e^	0.04 ^d^	0.03 ^c^	0.98
Sulfide	1	0.01 ^d^	0.01 ^cd^	0.01 ^bcd^	0.00 ^abc^	0.00 ^ab^	0.00 ^a^	0.00 ^bcd^	0.00 ^bcd^	0.00 ^abcd^	0.45
	2	0.01 ^f^	0.01 ^e^	0.01 ^d^	0.01 ^c^	0.01 ^b^	0.01 ^a^	0.01 ^d^	0.01 ^c^	0.01 ^c^	0.99
	15	0.07 ^d^	0.06 ^c^	0.06 ^bc^	0.05 ^ab^	0.05 ^ab^	0.05 ^a^	0.06 ^bc^	0.05 ^ab^	0.05 ^ab^	0.7
	50	0.04 ^e^	0.03 ^de^	0.03 ^de^	0.03 ^abc^	0.02 ^ab^	0.02 ^a^	0.03 ^cd^	0.03 ^bcd^	0.03 ^bcd^	0.72
	60	0.03 ^g^	0.03 ^fg^	0.03 ^ef^	0.02 ^bc^	0.02 ^b^	0.02 ^a^	0.03^def^	0.02 ^cde^	0.02 ^cd^	0.86
Desulfinyl	1	0.001 ^d^	0.001 ^d^	0.001 ^cd^	0.001 ^abc^	0.001 ^ab^	0.001 ^a^	0.001 ^bcd^	0.001 ^bcd^	0.001 ^bcd^	0.5
	2	0.002^c^	0.001^bc^	0.001 ^abc^	0.001 ^abc^	0.001 ^ab^	0.00 ^a^	0.001 ^abc^	0.001 ^abc^	0.001 ^abc^	0.41
	15	0.011 ^g^	0.010 ^f^	0.009 ^e^	0.008 ^bc^	0.008 ^b^	0.007 ^a^	0.009 ^e^	0.009 ^d^	0.008 ^c^	0.99
	50	0.006 ^f^	0.005 ^ef^	0.005 ^def^	0.004 ^abc^	0.004 ^ab^	0.004 ^a^	0.005 ^cde^	0.005 ^bcd^	0.005 ^bcd^	0.7
	60	0.005 ^f^	0.005 ^ef^	0.004 ^de^	0.004 ^bc^	0.003 ^b^	0.003 ^a^	0.004 ^de^	0.004 ^cd^	0.004 ^bcd^	0.82

*FL* Flooded, *ST* Saturated, *FC* Field Capacity, *WOM *Without organic amendment, *OM *Organic amendment, WD-herbicide application, *BDL* Below Detectable Limit

Mean of three replicate observations. In a column, means followed by a common letter are not significantly different (*P* < 0.05) by Duncan’s multiple range test

**Table 7:** Residue level of fipronil and its metabolites in paddy stem under different moisture regimes, organic amendment and herbicide

	DAA	Residue level (µg g^− 1^)									
		WOM			OM			WD			*R* ^2^
		FL	ST	FC	FL	ST	FC	FL	ST	FC	
Fipronil	1	0.128 ^d^	0.100 ^bc^	0.084 ^ab^	0.124 ^cd^	0.100 ^b^	0.076 ^a^	0.132 ^d^	0.104 ^b^	0.076 ^a^	0.74
	2	0.444 ^c^	0.424 ^abc^	0.408 ^ab^	0.432 ^bc^	0.420 ^abc^	0.404 ^a^	0.432 ^bc^	0.424 ^abc^	0.400 ^ab^	0.28
	15	0.032 ^g^	0.019 ^f^	0.012 ^c^	0.030 ^h^	0.017 ^d^	0.009 ^a^	0.032 ^h^	0.020 ^e^	0.011 ^b^	1.00
	50	0.006 ^f^	0.006 ^c^	0.003 ^a^	0.006 ^d^	0.008 ^b^	0.003 ^a^	0.007 ^e^	0.007 ^c^	0.003 ^a^	1.00
	60	BDL	BDL	BDL	BDL	BDL	BDL	BDL	BDL	BDL	-
Sulfone	1	BDL	BDL	BDL	BDL	BDL	BDL	BDL	BDL	BDL	-
	2	0.012 ^g^	0.010 ^d^	0.010 ^c^	0.009 ^e^	0.010 ^d^	0.008 ^a^	0.011 ^f^	0.010 ^d^	0.009 ^b^	0.98
	15	0.032 ^h^	0.031 ^e^	0.030 ^c^	0.027 ^f^	0.026 ^d^	0.025 ^a^	0.032 ^g^	0.030 ^d^	0.030 ^b^	0.99
	50	0.027 ^g^	0.024 ^e^	0.023 ^b^	0.027 ^f^	0.023 ^c^	0.021 ^a^	0.027 ^f^	0.027 ^d^	0.026 ^b^	1.00
	60	0.025 ^g^	0.024 ^d^	0.023 ^b^	0.023 ^e^	0.020 ^b^	0.020 ^a^	0.024 ^f^	0.020 ^c^	0.020 ^b^	1.00
Sulfide	1	BDL	BDL	BDL	BDL	BDL	BDL	BDL	BDL	BDL	-
	2	0.008 ^b^	0.007 ^a^	0.007 ^a^	0.007 ^a^	0.006 ^a^	0.007 ^a^	0.007 ^a^	0.007 ^a^	0.006 ^a^	0.99
	15	0.022 ^d^	0.021 ^cd^	0.020 ^abc^	0.019 ^cd^	0.018 ^bcd^	0.017 ^a^	0.022 ^d^	0.021 ^bcd^	0.020 ^ab^	0.48
	50	0.019 ^d^	0.016 ^cd^	0.016 ^ab^	0.019 ^cd^	0.016 ^abc^	0.015 ^a^	0.019 ^cd^	0.018 ^bcd^	0.018 ^ab^	0.49
	60	0.017 ^e^	0.017 ^c^	0.016 ^a^	0.016 ^c^	0.014 ^a^	0.014 ^a^	0.016 ^d^	0.014 ^b^	0.014 ^a^	0.99
Desulfinyl	1	BDL	BDL	BDL	BDL	BDL	BDL	BDL	BDL	BDL	-
	2	0.001 ^c^	0.001 ^ab^	0.001 ^ab^	0.001 ^bc^	0.001 ^ab^	0.001 ^a^	0.001 ^ab^	0.001 ^ab^	0.001 ^bc^	0.34
	15	0.003 ^c^	0.003 ^bc^	0.003 ^abc^	0.003 ^c^	0.003 ^bc^	0.003 ^a^	0.003 ^c^	0.003 ^bc^	0.003 ^ab^	0.43
	50	0.003 ^b^	0.003 ^b^	0.003 ^a^	0.003 ^b^	0.003 ^a^	0.002 ^a^	0.003 ^b^	0.003 ^b^	0.003 ^a^	0.60
	60	0.003 ^b^	0.002 ^ab^	0.002 ^a^	0.002 ^ab^	0.002 ^a^	0.002 ^a^	0.002 ^b^	0.002 ^ab^	0.002 ^a^	0.39

*FL* Flooded, *ST* Saturated, *FC* Field Capacity, *WOM *Without organic amendment, *OM *Organic amendment, WD-herbicide application, *BDL* Below Detectable Limit

Mean of three replicate observations. In a column, means followed by a common letter are not significantly different (*P* < 0.05) by Duncan’s multiple range test

**Table 8:** Residue level of fipronil and its metabolites in paddy leaf under different moisture regime, organic amendment and herbicide

	DAA	Residue level (µg g^− 1^)									
		WOM			OM			WD			*R* ^2^
		FL	ST	FC	FL	ST	FC	FL	ST	FC	
Fipronil	1	0.192 ^d^	0.068 ^a^	0.060 ^a^	0.144 ^b^	0.064 ^a^	0.052 ^a^	0.164 ^c^	0.068 ^a^	0.052 ^a^	0.97
	2	0.332 ^b^	0.312 ^ab^	0.304 ^ab^	0.324 ^ab^	0.312 ^ab^	0.288 ^a^	0.316 ^b^	0.308 ^ab^	0.304 ^ab^	0.25
	15	0.028 ^h^	0.024 ^e^	0.016 ^c^	0.017 ^f^	0.010 ^d^	0.008 ^a^	0.030 ^g^	0.025 ^d^	0.017 ^b^	1
	50	0.004 ^b^	0.004 ^b^	0.001 ^a^	0.004 ^b^	0.004 ^b^	0.001 ^a^	0.004 ^b^	0.004 ^b^	0.001 ^a^	1
	60	BDL	BDL	BDL	BDL	BDL	BDL	BDL	BDL	BDL	-
Sulfone	1	BDL	BDL	BDL	BDL	BDL	BDL	BDL	BDL	BDL	-
	2	0.009 ^d^	0.008 ^c^	0.008 ^b^	0.008 ^cd^	0.008 ^b^	0.007 ^a^	0.008 ^d^	0.008 ^b^	0.008 ^b^	0.96
	15	0.025 ^f^	0.0254 ^d^	0.024 ^c^	0.024 ^e^	0.024 ^c^	0.022 ^a^	0.015 ^e^	0.024 ^c^	0.024 ^b^	0.98
	50	0.023 ^f^	0.023 ^e^	0.023 ^b^	0.022 ^e^	0.020 ^c^	0.020 ^a^	0.023 ^f^	0.021 ^d^	0.020 ^ab^	1
	60	0.018 ^f^	0.017 ^d^	0.016 ^c^	0.017 ^e^	0.016 ^c^	0.016 ^a^	0.017 ^e^	0.017 ^d^	0.016 ^b^	0.98
Sulfide	1	BDL	BDL	BDL	BDL	BDL	BDL	BDL	BDL	BDL	-
	2	0.005 ^d^	0.004 ^c^	0.004 ^b^	0.004 ^c^	0.004 ^bc^	0.003 ^a^	0.005 ^c^	0.004 ^bc^	0.004 ^b^	0.76
	15	0.009 ^b^	0.009 ^b^	0.006 ^a^	0.008 ^b^	0.007 ^a^	0.006 ^a^	0.008 ^b^	0.008 ^b^	0.007 ^a^	0.74
	50	0.016 ^a^	0.016 ^a^	0.016 ^a^	0.015 ^a^	0.014 ^a^	0.014 ^a^	0.014 ^a^	0.014 ^a^	0.014 ^a^	0.16
	60	0.017 ^a^	0.016 ^a^	0.016 ^a^	0.016 ^a^	0.016 ^a^	0.016 ^a^	0.017 ^a^	0.017 ^a^	0.016 ^a^	0.06
Desulfinyl	1	BDL	BDL	BDL	BDL	BDL	BDL	BDL	BDL	BDL	-
	2	0.001 ^c^	0.001 ^bc^	0.001 ^b^	0.001 ^c^	0.001 ^bc^	0.001 ^b^	0.001 ^d^	0.001 ^c^	0.001 ^a^	0.76
	15	0.001 ^b^	0.001 ^b^	0.001 ^a^	0.001 ^b^	0.001 ^a^	0.001 ^a^	0.001 ^b^	0.001 ^b^	0.001 ^a^	0.7
	50	0.002 ^a^	0.002 ^a^	0.002 ^a^	0.002 ^a^	0.002 ^a^	0.002 ^a^	0.002 ^a^	0.002 ^a^	0.002 ^a^	0.16
	60	0.002 ^ab^	0.002 ^ab^	0.002 ^b^	0.002 ^ab^	0.002 ^ab^	0.002 ^ab^	0.002 ^ab^	0.002 ^ab^	0.002 ^a^	0.38

*FL* Flooded, *ST* Saturated, *FC* Field Capacity, *WOM *Without organic amendment, *OM *Organic amendment, WD-herbicide application, *BDL* Below Detectable Limit Mean of three replicate observations. In a column, means followed by a common letter are not significantly different (*P* < 0.05) by Duncan’s multiple range test

#### Persistence of total fipronil residue in whole paddy plant

**Fig. 3:**
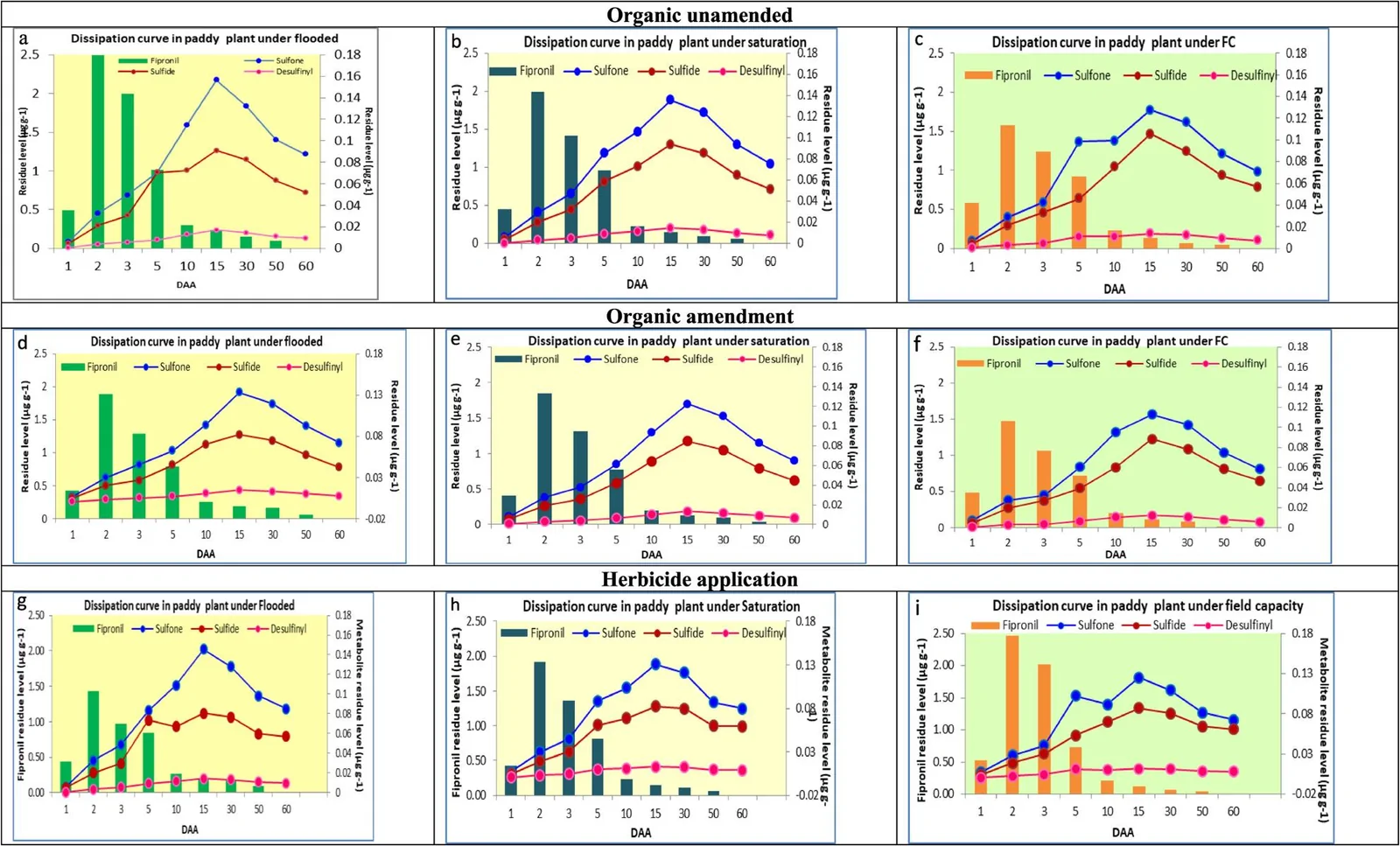
Dissipation of fipronil and its metabolites in paddy plant under different moisture regime with organic unamend (**a**, **b** ,**c** ), organic amended (**d**,**e**,**f**) and herbicide (**g**,**h**,**i**) treatment

Figure 3 illustrates the dissipation pattern of total fipronil residues (parent compound and metabolites) in paddy plants under different moisture regimes, organic amendment, and herbicide treatments. Total residues were highest under flooded conditions, followed by saturated and field capacity soils. Unamended treatments recorded higher residues than organic-amended treatments, while herbicide-treated soils showed intermediate residue levels. Residues increased from 0.59 µg g⁻¹ at 1 DAA to a peak of 2.55 µg g⁻¹ at 2 DAA under flooded unamended conditions, followed by steady decline to 0.02 µg g⁻¹ at 70 DAA. Above-ground residues decreased to 0.09 µg g⁻¹ by 50 DAA, whereas root residues remained comparatively higher (0.09–0.17 µg g⁻¹). Table 9.

**Table 9:** Residue level in different paddy substrates under different moisture regimes with organic amendment and herbicide application

		Residue level (µg g^− 1^)								
		WOM			OM			WD		
Plant parts	DAA	FL	ST	FC	FL	ST	FC	FL	ST	FC
Root	1	0.27	0.24	0.24	0.3	0.26	0.26	0.37	0.3	0.32
Straw		0.13	0.13	0.12	0.08	0.1	0.1	0.08	0.1	0.08
Leaf		0.19	0.16	0.14	0.06	0.07	0.06	0.05	0.07	0.05
Total		0.59	0.54	0.51	0.44	0.44	0.43	0.5	0.46	0.45
Root	5	0.82	0.73	0.63	0.46	0.55	0.43	0.7	0.63	0.62
Straw		0.18	0.21	0.22	0.23	0.18	0.21	0.17	0.21	0.16
Leaf		0.15	0.18	0.22	0.2	0.14	0.19	0.14	0.16	0.13
Total		1.16	1.12	1.08	0.89	0.88	0.83	1.01	0.99	0.9
Root	10	0.38	0.34	0.25	0.2	0.19	0.23	0.31	0.25	0.26
Straw		0.07	0.07	0.11	0.11	0.12	0.08	0.07	0.1	0.09
Leaf		0.05	0.05	0.06	0.05	0.05	0.05	0.04	0.06	0.06
Total		0.5	0.46	0.42	0.36	0.37	0.36	0.42	0.41	0.41
Root	50	0.17	0.16	0.14	0.11	0.11	0.09	0.13	0.13	0.11
Straw		0.05	0.06	0.05	0.05	0.04	0.04	0.05	0.05	0.05
Leaf		0.04	0.05	0.04	0.05	0.04	0.04	0.04	0.04	0.04
Total		0.27	0.27	0.23	0.2	0.18	0.16	0.22	0.22	0.21
Root	70	BDL	BDL	BDL	BDL	BDL	BDL	BDL	BDL	BDL
Straw		BDL	BDL	BDL	BDL	BDL	BDL	BDL	BDL	BDL
Leaf		0.018	0.017	0.016	0.017	0.015	0.015	0.017	0.016	0.015
Total		0.018	0.017	0.016	0.017	0.015	0.015	0.017	0.016	0.015

Notation: Average residue level of three replication and within ± 0.02 standard deviation

#### Absorption and root uptake of fipronil in paddy: Root bio concentration factor (RCF) 

The Root Bioconcentration Factor (RCF) is used to assess the absorption capacity of fipronil by paddy roots. It is calculated as the ratio of pesticide concentration in roots to its concentration in soil. RCF values ranged from 0.14 to 0.50 at 1 DAA and increased to peak levels at 3 DAA, ranging from 0.45 (OM + FC) to 2.60 (WOM + FL), indicating maximum root uptake during the early stage after application (Table 10). Subsequently, RCF values declined during 5–15 DAA, ranging from 0.17 to 0.49, and later increased at 60 DAA to 0.31–0.81. Higher RCF values were consistently observed in flooded unamended soils, whereas lower values were recorded under field capacity and organic-amended conditions. Organic amendment reduced RCF values compared to unamended soils, while herbicide-treated soils showed intermediate values across all moisture regimes.

**Table 10:** Root bio concentration factor in paddy under different moisture regimes with organic amendment and herbicide application

Residue level (µg g^− 1^)								
DAA	1	3	5	10	15	30	50	60
T_1_	0.50	2.60	2.32	1.49	0.49	0.51	0.46	0.81
T_2_	0.27	1.18	0.86	0.76	0.29	0.41	0.39	0.65
T_3_	0.23	1.03	0.77	0.61	0.21	0.23	0.20	0.38
T_4_	0.29	1.27	0.94	0.83	0.39	0.37	0.37	0.67
T_5_	0.16	0.50	0.37	0.34	0.23	0.30	0.27	0.46
T_6_	0.14	0.45	0.33	0.35	0.17	0.22	0.20	0.31
T_7_	0.39	2.19	1.89	1.12	0.42	0.46	0.49	0.64
T_8_	0.19	0.90	0.68	0.60	0.26	0.32	0.31	0.47
T_9_	0.16	0.56	0.47	0.45	0.18	0.22	0.18	0.29

**Table 11:** Translocation factor in paddy under different moisture regimes with organic amendment and herbicide application

Residue level (µg g^− 1^)								
DAA	1	3	5	10	15	30	50	60
T_1_	0.87	0.45	0.42	0.53	0.50	0.39	0.38	0.56
T_2_	0.57	0.63	0.64	0.59	0.56	0.41	0.42	0.56
T_3_	0.53	0.65	0.63	0.56	0.46	0.51	0.52	0.63
T_4_	0.89	0.62	0.65	0.60	0.54	0.51	0.51	0.69
T_5_	0.63	0.99	1.01	0.76	0.61	0.46	0.58	0.86
T_6_	0.54	0.97	0.90	0.65	0.53	0.51	0.61	0.93
T_7_	0.93	0.45	0.41	0.56	0.55	0.47	0.43	0.72
T_8_	0.65	0.63	0.65	0.59	0.55	0.54	0.53	0.88
T_9_	0.53	0.88	0.75	0.54	0.54	0.56	0.61	0.87

#### Translocation and distribution of fipronil in paddy plant: Tanslocation factor (TF) 

 After fipronil in the soil is absorbed by rice roots, it may be translocated upwards to the aboveground parts of the plant. Therefore, the Translocation Factor (TF), defined as the ratio of pesticide concentration in shoots to that in roots, was used to assess the rice plant’s ability to transport pesticides from roots to shoots. The result found the TF value (Table 11) was gradually decline from the initial day upto 30DAA across all the treatment, indicating reduced translocation over time. But there was gradual increase in TF value after 30DAA reaching the TF value from 0.56(WOM + FL) to 0.93(OM + FC).

#### Fipronil residues in paddy plant at harvest

Paddy plant samples at harvest, including roots, stem, leaves, and whole grain, were analyzed for fipronil and its metabolite residues. In all paddy substrates, the residues of fipronil and its metabolites were below the detectable limit of 0.01 mg kg⁻¹ (Table 12).

**Table 12:** Residue level in paddy plant at harvest

Matrices	Residue level (µg g^− 1^)								
	WOM			OM			WD		
	FL	ST	FC	FL	ST	FC	FL	ST	FC
Grain	0.002–0.028	0.002–0.020	0.002–0.027	0.002–0.014	0.002–0.010	0.002–0.006	0.002–0.014	0.002–0.010	0.002–0.006
Straw	0-0.023	0-0.021	0-0.020	0-0.024	0-0.020	0-0.020	0-0.015	0-0.015	0-0.013
Soil	0-0.021	ND	0-0.022	0-0.011	ND	0-0.012	0-0.04	0-0.06	0-0.12

#### Risk assessment

Potential risk due to the presence of fipronil residues in paddy ecosystem was predicted for human, animal by calculating Dietary risk quotient (RQd). The current available Maximum Residue Limits (MRLs) of fipronil in rice are 0.01 [12] and 0.005 [13] µg g^-1^ or mg kg^-1^for India and European countries, accordingly.

At harvest, all paddy plant parts (roots, stem, leaves, and whole grain) showed fipronil and metabolite residues below the MRL (0.01 µg g⁻¹ or mg/kg), indicating no residue-related risks. The dietary intake risk assessment for fipronil indicates an acceptable daily intake (ADI) of 0.0002 mg/kg body weight [12]. In India, with an assumed average adult body weight of 65 kg, the ADI for an individual is calculated to be 0.013 mg (0.0002 mg/kg bw × 65 kg body weight). According to the dietary guidelines in India [14], the average daily rice consumption is 0.260 kg per person. Based on the highest total residue of 0.02 mg/kg of fipronil detected in harvested rice grain, the estimated dietary intake of fipronil per person would be approximately 0.0052 mg (0.260 kg × 0.02 mg/kg). This value is significantly lower than the ADI of 0.013 mg, resulting in an acute risk quotient of only 40%.

Table 13 depicts the estimated dietary intake (EDI) and Dietary risk quotient (RQd) at 50DAA and at harvest stage. The Table shows that fipronil residues in paddy straw at 50 DAA were above MRL and paddy grain at harvest were well below the MRL, with Estimated Daily Intake (EDI) values extremely low. Dietary Risk Quotient (RQd) values at 50 DAA ranged from 0.42 to 1.47, while at harvest they dropped to 0.36–0.94, indicating reduced dietary risk over time. Organic amendments under FC moisture regime consistently showed the lowest residues (0.01 µg g⁻¹) and RQd (0.36), whereas unamended and herbicide under FL moisture regime recorded the highest values (0.94 and 0.84 respectively). All RQd values were < 2, suggesting no significant human health risk.

**Table 13:** Dietary risk quotient (RQd) of total fipronil residues in paddy grain for human and cattle under different moisture regimes with organic amendment and herbicide application

Treatments	50DAA			Harvest stage					
	Straw			Straw			Grain		
	Residue(µg g^− 1^)	EDI	RQd	Residue(µg g^− 1^)	EDI	RQd	Residue (µg g^− 1^)	EDI	RQd
T_1_	0.06	0.0003	1.47	0.03	0.0010	0.35	0.04	0.0002	0.94
T_2_	0.05	0.0002	1.18	0.02	0.0009	0.31	0.03	0.0001	0.73
T_3_	0.02	0.0001	0.57	0.02	0.0008	0.29	0.02	0.0001	0.47
T_4_	0.04	0.0002	0.92	0.02	0.0009	0.32	0.03	0.0001	0.74
T_5_	0.04	0.0002	1.06	0.02	0.0008	0.29	0.03	0.0001	0.72
T_6_	0.02	0.0001	0.42	0.02	0.0008	0.26	0.01	0.0001	0.36
T_7_	0.05	0.0003	1.3	0.02	0.0009	0.32	0.03	0.0002	0.84
T_8_	0.04	0.0002	0.96	0.02	0.0009	0.30	0.03	0.0001	0.73
T_9_	0.02	0.0001	0.6	0.02	0.0008	0.28	0.02	0.0001	0.44

Notation: DAA = Days after application; Harvest stage= 70DAA; Total residue (µgg 1) = Detected concentration value [in case where the residue was not detectable, ½ LOQ value (i.e., 0.0025 µgg 1)of fipronil and its metabolites was considered]; EDI = Estimated Daily Intake (mg kg 1 body weight)

## Discussion

The high linearity obtained for fipronil and its metabolites confirmed the suitability of the LC–MS/MS method for quantitative residue analysis in paddy soil and plant matrices. Acceptable recovery percentages and RSD values which were within the acceptable limit of < 20% demonstrated good accuracy and precision of the method, while minimal matrix effects (within ± 10%) indicated negligible ion suppression or enhancement during LC–MS/MS analysis. All validation parameters complied with the criteria prescribed in [10], confirming the robustness and suitability of the method for routine analysis of fipronil and its metabolites in paddy samples.

The half-life in non-cropped soil (20.82 days) was approximately 75% longer than in flooded unamended cropped soil (11.87 days), demonstrating the contribution of plant uptake and rhizosphere activity to fipronil dissipation [15]. Fipronil residues declined below detectable limits by 50 days after application (DAA) in cropped soils, whereas non-cropped soils retained residues above the maximum residue limit (MRL − 0.01 µg g⁻¹ or mg/kg) until 60 DAA [14]. This difference underscores the crucial role of crop-soil interactions and rhizosphere microbial activity in accelerating pesticide degradation [16].

Although fipronil was applied uniformly across all treatments at the recommended dose, the residue concentrations reported at 0 DAA were determined after the respective moisture regimes and treatment conditions had been established and allowed to equilibrate. Therefore, differences in initial residue concentrations reflected treatment-specific effects on pesticide distribution, sorption, and extractability under varying soil moisture and organic amendment conditions, rather than application rate.

### Moisture regime effects on fipronil dissipation

The pronounced influence of soil moisture on fipronil persistence was clearly demonstrated through the differential dissipation patterns observed across the three moisture regimes. Under different moisture regimes, distinct redox conditions were observed. Flooded soils exhibited strongly negative Eh values (− 95 to − 122 mV), indicating highly reduced conditions. In contrast, saturated soils showed moderately positive Eh values (100–150 mV), reflecting mildly reducing conditions. Soils maintained at field capacity recorded higher positive Eh values (200–300 mV), indicative of oxidizing conditions.

Flooded soils showed the shortest half-life (11.87 days), which was approximately 14% lower than field capacity soil with organic amendment (13.75 days), indicating faster dissipation under reduced conditions because the strongly reduced redox conditions (− 95 to − 122 mV) promoted reductive degradation and microbial transformation of fipronil [9]. This finding aligns with previous observations by [15], who demonstrated that higher soil moisture (40% water holding capacity) accelerated degradation of parent compounds and enhanced metabolic transformation efficiency. Similarly, biochar amendments under high moisture conditions promoted metabolite formation.

The higher persistence observed under field capacity conditions resulted from oxidizing soil conditions (200–300 mV), which reduced reductive degradation and slowed microbial transformation compared to flooded soils. Saturated soils displayed intermediate behavior with redox potentials around 100–150 mV, representing mildly reducing conditions.

### Role of organic amendment and pesticide interactions

The effect of organic amendment on fipronil persistence varied with the prevailing moisture regime. The difference in degradation arises because the influence of organic amendment was moisture-dependent. Under flooded conditions, dissolved organic matter increased the aqueous-phase availability of fipronil, enhancing microbial degradation and dissipation by 8%. However, under saturated and field-capacity conditions, organic matter increased sorption of fipronil to soil organic constituents, reducing its availability for degradation and plant uptake, thereby increasing persistence. This dual behavior supports the findings of [8], who demonstrated that organic amendments can both enhance persistence via sorption and promote dissipation by increasing bioavailability, depending on soil moisture and environmental conditions.

Herbicide application demonstrated complex interactions with fipronil dissipation patterns. Herbicide promoted more rapid dissipation compared to organic-amended soils, suggesting that pesticide-pesticide interactions [17] reduced fipronil sorption to soil particles. However, dissipation remained slower than in unamended soils. This observation is supported by our previous study [18], which demonstrated that herbicide application altered the structure and functional potential of soil microbial communities, thereby reducing microbial populations. The altered microbial activity contributed to reduced biodegradation efficiency and consequently greater persistence of fipronil in herbicide-treated soils [17]., who emphasized that molecular properties of pesticides including solubility, ionizability, hydrophilicity, and lipophilicity, combined with soil properties, are critical determinants of pesticide behavior in soil environments. The continuous application of multiple pesticides in agricultural fields creates complex residue interactions that influence individual pesticide fate [17].

### Metabolite formation and transformation pathways

The metabolite distribution patterns provided clear evidence for the influence of redox conditions on fipronil transformation pathways. Although parent fipronil residues declined progressively over time, fipronil sulfone accumulated as the predominant metabolite, followed by fipronil sulfide and desulfinyl derivatives. Higher sulfone residues (0.14 µg g⁻¹ in FC soil @ 70DAA) under field capacity and saturated conditions resulted from oxidizing environments that favored conversion of fipronil to sulfone indicated greater stability and slower degradation in oxygen-rich environments [18]. This apparent increase in fipronil sulfone relative to the parent compound is attributable to the continuous oxidative transformation of fipronil coupled with the greater persistence of fipronil sulfone in soil. Consequently, sulfone concentrations may temporarily exceed the residual parent concentration at later sampling intervals, reflecting metabolite accumulation rather than enhanced formation rates. Similar observations have been reported in previous studies [19] where fipronil sulfone was identified as the dominant and more persistent degradation product under aerobic soil conditions.

Conversely, flooded soils exhibited lower sulfone levels but higher fipronil sulfide accumulation (0.10 µg g⁻¹ at 15 DAA), reflecting the dominance of reductive pathways under anaerobic conditions. This inverse relationship between sulfone and sulfide formation clearly demonstrates the strong influence of redox status on fipronil transformation, supporting observations by [20], who found that low redox potential in submerged soils promotes reduction of fipronil to its sulfide form.

Desulfinyl metabolite concentrations remained below 0.01 µg g⁻¹ in all treatments but were comparatively Higher in field capacity soils suggest that greater surface exposure under non-flooded conditions promoted photodegradation [21].

Fipronil degrades in soil through oxidation, reduction, hydrolysis, and photolysis, producing multiple metabolites. This study focused on fipronil sulfone, sulfide, and desulfinyl, the major environmental metabolites commonly considered in risk assessments [21]. Although other metabolites, such as fipronil amide and transient intermediates, have been reported, they generally occur at lower concentrations and were excluded due to the unavailability of certified reference standards. Thus, while major degradation pathways were represented, minor products cannot be excluded. Future high-resolution mass spectrometry studies could provide a more comprehensive characterization of fipronil transformation in tropical paddy soils.

### Degradation kinetics and half-life analysis

The kinetic analysis confirmed that fipronil degradation followed first-order kinetics (Fig. 2) across all treatment combinations, consistent with findings by [21]. The shortest half-life (11.87 days) occurred in flooded unamended soil with the highest dissipation rate constant (k = 0.058), indicating optimal conditions for rapid degradation under anaerobic conditions. The longest persistence observed in field capacity soil with organic amendment (t₁/₂ = 13.75 days) reflects the combined effects of reduced microbial activity under drier conditions and enhanced adsorption of fipronil residues onto organic matter, which reduces bioavailability for degradation [19]. Laboratory incubation studies by [7] under controlled conditions have shown comparatively longer persistence ranging from 122 to 128 days.

### Plant uptake and translocation dynamics

Fipronil uptake and distribution within rice plants followed predictable patterns based on the systemic nature of the insecticide [21]. The distribution pattern of fipronil residues in paddy plants followed the order root > stem > leaf throughout the experimental period. At peak accumulation (2 DAA), root residues were 3–4 times higher than stem residues and 5–6 times higher than leaf residues, indicating limited upward translocation of fipronil from roots to aerial plant parts. This trend confirms that roots acted as the primary accumulation site, while translocation to stem and leaf tissues remained comparatively low resulted from the hydrophobic nature of fipronil, which restricted upward translocation within rice plants. Residue accumulation was consistently higher under flooded conditions compared to saturate and field capacity regimes, while organic amendment reduced overall plant residue levels, indicating decreased bioavailability [16]. Flooding increased the dissolved fraction and mobility of fipronil in soil solution, resulting in greater initial uptake by rice roots.

Root Bioconcentration Factor (RCF) analysis indicated maximum fipronil uptake during 2–5 DAA, corresponding to peak pesticide availability in soil, followed by a gradual decline due to degradation and translocation within plant tissues. The increase in RCF after 15 DAA occurred because declining soil residues combined with continued retention of fipronil in root tissues increased the root-to-soil concentration ratio. RCF values in unamended soils were 17.2% higher than in organic-amended soils indicating greater pesticide availability in the absence of organic matter. These findings are consistent with previous reports showing positive relationships between root bioconcentration and pesticide degradation rate and molecular weight, but negative relationships with water solubility [22].

Translocation Factor (TF) values decreased from 0.45 to 0.99 at 2 DAA to 0.39–0.46 at 30 DAA, indicating restricted upward movement of fipronil over time due to degradation and its hydrophobic nature, which favors accumulation in roots over aerial plant parts. The increase in TF after 30 DAA resulted from rapid decline of root residues, causing even small shoot residues to produce a higher shoot-to-root ratio. Similar trends have been reported for hydrophobic pesticides in crop systems [23–26]. The observed dissipation behavior and half-life values were comparable with earlier studies in grape, chili, cauliflower, groundnut, and sugarcane systems [27–31].

### Food safety and risk assessment

The food safety assessment demonstrated that fipronil application at the recommended dose (75 g a.i. ha⁻¹) poses minimal risk to human health under the studied conditions. All paddy plant parts showed total residues below the Maximum Residue Limit (0.01 µg g⁻¹) at harvest, with Dietary Risk Quotient (RQd) values ranging from 0.36 to 0.94, all well below the safety threshold of 2. These findings are consistent with studies by [13&24], who reported an acute risk quotient of 0.65% for fipronil use in peanut crops, and [4], who observed 92–96% degradation of fipronil residues within 15 days with no significant human health risk.

The estimated dietary intake of fipronil (0.0052 mg per person per day) was significantly lower than the acceptable daily intake (0.013 mg), resulting in an acute risk quotient of only 40%. Organic amendment under field capacity conditions consistently produced the lowest residue levels and risk quotients, suggesting this management combination offer optimal safety margins while maintaining pest control efficacy.

### Implications for agricultural practice

The findings of this study have important implications for sustainable rice production in tropical regions. Maintaining flooded conditions after fipronil application along with the addition of organic amendments can help farmers reduce pesticide persistence in soil and minimize residue accumulation in rice plants under tropical paddy cultivation. The demonstrated safety of fipronil at recommended application rates, combined with the 14% shorter half-life observed under flooded conditions, supports its continued use in paddy systems. The beneficial effects of organic amendment in reducing plant residue accumulation while maintaining pest control efficacy suggest that integrated organic matter management can optimize pesticide use efficiency.

The period of maximum pesticide effectiveness (2–15 DAA) identified through bioconcentration factor analysis provides valuable guidance for timing pest management interventions. The metabolite persistence patterns indicate that the combined action of parent compound and metabolites provides extended pest control, supporting the practical utility of fipronil in integrated pest management programs.

### Study limitations and future research

This study focused on a single application rate under controlled field conditions in one geographic location. Future research should investigate the effects of multiple applications, different application rates, and varying climatic conditions on fipronil fate and behavior. Long-term studies examining the cumulative effects of repeated fipronil applications on soil microbial communities and ecosystem health would provide valuable insights for sustainable pesticide management. Additionally, investigation of fipronil behavior in different soil types and rice varieties would enhance the generalizability of these findings.

## Conclusions

Fipronil dissipation in tropical paddy soil is significantly influenced by moisture conditions, organic matter content, and redox potential. Flooded conditions accelerated degradation, resulting in the shortest half-life (11.87 days), whereas field capacity with organic amendment showed greater persistence (t₁/₂ = 13.75 days). Rhizosphere-associated cropped soils showed rapid residue decline below detectable levels by 50 DAA, whereas non-cropped soils exhibited prolonged persistence beyond the MRL up to 60 DAA. Oxidizing conditions favored formation of fipronil sulfone, which persisted up to 70 DAA (0.14 µg g⁻¹). Organic amendment reduced residue accumulation and translocation to rice plants, with all residues remaining below the MRL at harvest and dietary risk quotient values below 1 indicating minimal human health risk at the recommended dose of 75 g a.i. ha⁻¹. Flooded conditions combined with organic amendment provided the most effective balance between rapid dissipation and reduced residue transfer, supporting safe and sustainable fipronil use in tropical paddy cultivation. The study provides important agronomic and regulatory evidence supporting safe fipronil application in tropical rice production systems, while emphasizing the need for continuous monitoring of persistent metabolites and their effects on soil microbial ecology.

## Data Availability

Data can be made available on request through email to first author.
